# Purple urine bag syndrome: report of an unusual case in a patient with a chronic urinary catheter

**DOI:** 10.17843/rpmesp.2026.431.15050

**Published:** 2026-02-21

**Authors:** Jorge Luis Hurtado-Alegre, Jhonatan Mauricio Crispin-Ayala, Jorge del Piero Alvarez-Canto, Jhosef Franck Quispe-Pari

**Affiliations:** 1 Universidad Continental, Huancayo, Peru.; 2 Infectology Service, Hospital Nacional Ramiro Priale, Huancayo, Peru.; 3 Universidad Nacional del Centro del Peru, Huancayo, Peru.

**Keywords:** Urinary Catheters, Bacteriuria, Asymptomatic Infections, Klebsiella pneumoniae

## Abstract

Purple urine bag syndrome (PUBS) is an infrequent and generally asymptomatic condition characterized by the purple coloration of urine in patients with a urinary catheter, secondary to bacterial metabolism of tryptophan and the production of pigments during the process. We describe the case of an 82-year-old male with a history of benign prostatic hyperplasia, arterial hypertension, and long-term Foley catheter use, who presented for a preoperative consultation without symptoms, exhibiting purple urine of 48 hours’ duration. The urine culture showed growth of extended-spectrum beta-lactamase-producing *Klebsiella pneumoniae*. Given the absence of urinary or systemic symptoms, it was decided not to administer antibiotics and to only replace the urinary drainage system. The patient progressed favorably, with resolution of the color change. PUBS, although striking, is a benign finding associated with asymptomatic bacteriuria, and its management should avoid unnecessary antibiotic use.

## INTRODUCTION

Purple Urine Bag Syndrome (PUBS) is a rare clinical entity characterized by a striking purple discoloration of the urinary drainage system, typically observed in patients with long-term indwelling urinary catheters. Although visually alarming, this phenomenon is usually benign and asymptomatic in most cases; however, it is often associated with urinary tract infections (UTIs) and underlying debilitating conditions that increase patient morbidity ^1^^-^^3^.

It was first described in 1978, although similar clinical observations have been suggested since the 19th century, as in the case of King George III ^2^^,^^3^. The characteristic pigmentation results from the production of compounds derived from bacterial metabolism of tryptophan, making PUBS a visible manifestation of a complex underlying biochemical process ^1^^,^^3^^,^^4^.

Recent studies indicate that PUBS may have a significant prevalence in certain patients. It has been reported in up to 9.8% of patients with long-term urinary catheterization ^2^^,^^3^. In geriatric hospitals, up to 27% of patients with dementia and indwelling urinary catheters developed this condition ^(^^1^. Frequently associated risk factors include female sex, advanced age, dementia, chronic constipation, dehydration, chronic kidney disease, alkaline urine, and a high bacterial load in the urine ^(1-3)^. This condition predominantly affects women and individuals with overall debilitated states, which may be explained by both anatomical factors and the higher frequency of chronic catheterization in these populations ^2^^,^^3^^,^^5^.

This case is interesting due to its presentation in an asymptomatic male patient colonized with extended-spectrum beta-lactamase (ESBL)-producing *Klebsiella pneumoniae*, as it highlights the benign nature of the syndrome ^6^ and the importance of conservative management without the use of antibiotics.

## CASE REPORT

An 82-year-old male patient attended to a medical facility with a history of benign prostatic hyperplasia (BPH), arterial hypertension controlled with losartan 50 mg/day, and long-term use of a silicone Foley urinary catheter, size 18 Fr, for approximately three years. He had no history of drug allergies or recent antibiotic use. He reported occasional use of osmotic laxatives for functional constipation. The patient maintained an approximate daily fluid intake of 1.5 L and lived at home under family care.

**Figure 1:**
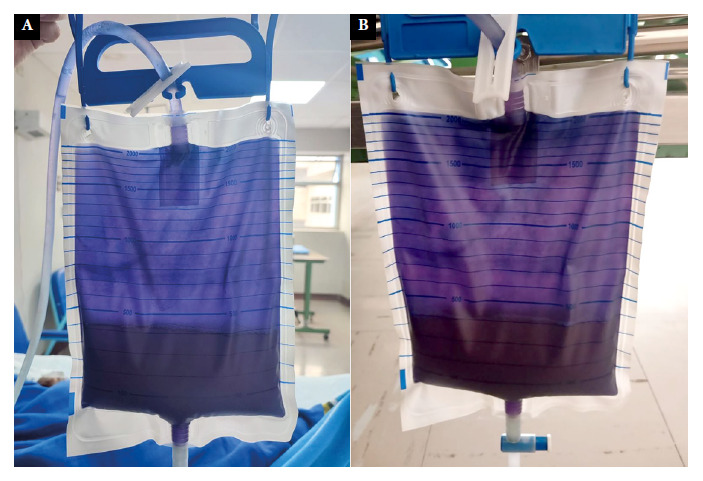
Presence of purple discoloration of the urine contained in the collection bag. (A) Bag collected during the first day of hospitalization; (B) bag collected during the third day of hospitalization, prior to the replacement of the drainage system.

His chief complaint was the observation of a purple discoloration of the urine during the past 48 hours. The patient denied fever, dysuria, suprapubic pain, or flank pain. On physical examination, he had a normal temperature (36.7 °C), had a blood pressure of 122/68 mmHg, a heart rate of 76 bpm, a respiratory rate of 18 breaths per minute, and oxygen saturation of 91% on room air, without signs of bladder irritation or systemic involvement. A purple discoloration of both the urinary catheter and the collection bag was observed (figure 1), a finding consistent with Purple Urine Bag Syndrome (PUBS).

Urinalysis showed an alkaline pH of 8.5, positive nitrites, positive leukocyte esterase, increased leucocytes, and abundant bacteria. Renal function was preserved (serum creatinine 1.0 mg/dL, estimated clearance > 60 mL/min). A urine sample was obtained for analysis and culture. Urine culture revealed growth of extended-spectrum beta-lactamase (ESBL)-producing *Klebsiella pneumoniae*. Despite these microbiological findings, the patient remained asymptomatic throughout follow-up.

## MANAGEMENT

In the absence of urinary or systemic symptoms, antibiotics were withheld in accordance with current guidelines for the management of asymptomatic bacteriuria. The patient was admitted and a complete replacement of the urinary drainage system was performed using standard aseptic techniques, including the exchange of both the Foley catheter and the collection bag. In addition, the patient received education on preventive measures for infection, such as proper perineal hygiene, constipation management, maintenance of adequate urinary flow, and scheduled system replacement every three weeks.

### Evolution and follow-up

**Figure 2:**
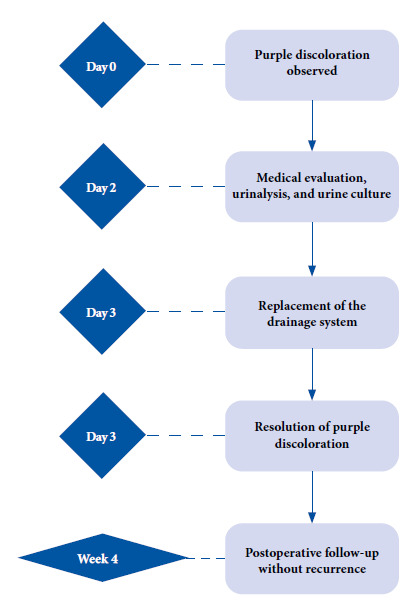
Flowchart of case progression and management.

Figure 2 presents a timeline of events. The patient subsequently underwent the scheduled surgical procedure without complications. During the postoperative period and subsequent follow-up visits, no recurrence of the purple discoloration in the urine or collection bag was observed.

## DISCUSSION

**Figure 3:**
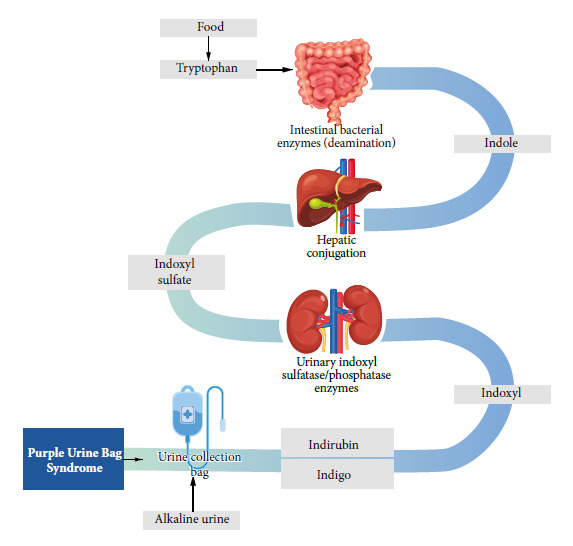
Pathophysiology of Purple Urine Bag Syndrome. It illustrates how dietary tryptophan is transformed by intestinal bacteria and subsequently metabolized in the liver into a compound excreted in the urine. In the presence of certain bacteria and alkaline urine, pigments are formed that react with the material of the urine collection bag, producing the characteristic purple color.

Source: own elaboration.

The pathophysiology of Purple Urine Bag Syndrome (PUBS) (figure 3) is based on a complex metabolic cascade of tryptophan. This essential amino acid is converted into indole by the intestinal microbiota; subsequently, indole is absorbed through the portal circulation and metabolized in the liver to 3-hydroxyindole by cytochrome P450 2E1 (CYP2E1), which is then sulfonated by the hepatic isoform SULT1A1 to indoxyl sulfate ^7^^,^^8^. This metabolite, excreted through the urine, can be degraded in the urinary tract by bacterial phosphatases and sulfatases from *Providencia stuartii*, *Proteus mirabilis*, *Escherichia coli*, *Pseudomonas aeruginosa*, *Klebsiella pneumoniae*, *Morganella* spp., and *Citrobacter* spp. in an alkaline environment, generating indigo (blue) and indirubin (red). The combination of these pigments in polyvinyl chloride (PVC) urine collection bags results in the characteristic purple discoloration ^7^^-^^9^. It should be noted that not all bacterial strains possess the same enzymatic activity; therefore, the presence of bacteriuria alone does not directly lead to the development of the syndrome ^8^^,^^9^. Additionally, elevated concentrations of indoxyl sulfate in patients with renal insufficiency, due to reduced clearance, increase substrate availability for pigment formation ^8^^,^^10^.

Among the most commonly associated risk factors for PUBS are female sex, advanced age, chronic constipation, dementia, immobility, chronic kidney disease, a tryptophan-rich diet, long-term indwelling urinary catheterization, and alkaline urine ^11^^-^^14^. Female urinary anatomy and impairments in self-care and hygiene favor bacterial colonization and recurrent urinary tract infections, particularly in institutionalized or bedridden patients ^9^^-^^11^. Alkaline urine is a key, though not indispensable, factor and is often induced by urease-producing bacteria such as *Proteus mirabilis*, *Providencia stuartii/rettgeri*, *Escherichia coli*, *Klebsiella pneumoniae*, and *Pseudomonas aeruginosa*, all frequently identified in urine cultures of patients with PUBS ^7^^,^^8^. Furthermore, constipation slows intestinal transit and prolongs exposure of tryptophan to intestinal flora, facilitating its conversion to indole and, consequently, the production of indoxyl sulfate ^7^^,^^11^^,^^13^. Similarly, a high urinary bacterial load has been correlated with a higher incidence of the syndrome ^8^^,^^9^^,^^12^.

Management of PUBS is based on addressing predisposing factors rather than treating the discoloration itself, as it is generally a benign condition, and, in most cases, the pigmentation resolves after correction of the underlying cause ^7^^,^^8^^,^^10^. Nevertheless, proper management of urinary devices is essential, including the replacement of the collection bag and catheter, as well as the implementation of hygiene measures and preventive care to avoid further bacterial colonization ^7^^,^^9^^,^^12^. In patients with comorbidities such as chronic kidney disease, monitoring of indoxyl sulfate levels may be relevant, due to its association with progression of nephropathy and cardiovascular events ^8^^,^^12^. In severe cases with infectious complications or signs of sepsis, early initiation of appropriate antibiotic therapy based on risk factors for bacterial resistance or according to antibiogram results has been shown to reduce associated morbidity and mortality ^8^^,^^14^.

In line with the guidelines of the Infectious Diseases Society of America (IDSA) ^15^ and the United States Preventive Services Task Force (USPSTF) ^(^^16^, bacteriuria in patients with indwelling urinary catheters should not be treated with antibiotics in the absence of symptoms, thereby avoiding antimicrobial resistance and adverse effects. These principles are also promoted by the Centers for Disease Control and Prevention (CDC) ^17^ and the National Institute for Health and Care Excellence (NICE) ^18^, which emphasize reducing unnecessary antimicrobial use and preventing resistance.

In conclusion, we present a case which illustrates that Purple Urine Bag Syndrome (PUBS), despite its alarming appearance, is a benign and generally self-limited condition associated with asymptomatic bacteriuria in patients with chronic indwelling urinary catheters. In this context, the identification of extended-spectrum beta-lactamase (ESBL)-producing *Klebsiella pneumoniae* in the absence of clinical manifestations of urinary tract infection reinforces the importance of distinguishing bacterial colonization from true infection, thereby avoiding unnecessary therapeutic interventions.

The complete resolution of the condition following replacement of the urinary drainage system using standard aseptic technique, along with correction of risk factors, implementation of hygiene measures, and patient education, demonstrates that conservative management is sufficient in the absence of urinary or systemic symptoms.

This approach is consistent with the recommendations of the IDSA, USPSTF, CDC, and NICE, which emphasize the restriction of antibiotic use only to symptomatic cases, thus promoting rational antimicrobial use and the prevention of bacterial resistance. Overall, this case highlights the importance of clinical judgment, appropriate patient monitoring, and best practices in urinary catheter management as key elements for optimizing clinical outcomes and preventing complications.
