# Supplementary material for: Purple urine bag syndrome: report of an unusual case in a patient with a chronic urinary catheter
Source: Rev Peru Med Exp Salud Publica. 2026 Feb 21;43(1):138–42. doi: 10.17843/rpmesp.2026.431.15050 (PMC13245986; doi:10.17843/rpmesp.2026.431.15050)
Supplement: Translation

# Translation: Síndrome de la bolsa púrpura: reporte de un caso inusual en un paciente con sonda vesical crónica

**Authors:** Jorge Luis Hurtado-Alegre, Jhonatan Mauricio Crispin-Ayala, Jorge del Piero Alvarez-Canto, Jhosef Franck Quispe-Pari

**Affiliations:** 1 Universidad Continental, Huancayo, Perú.; 2 Infectology Service, Hospital Nacional Ramiro Priale, Huancayo, Perú.; 3 Universidad Nacional del Centro del Peru, Huancayo, Perú.

**Keywords:** Catéteres Urinarios, Bacteriuria, Infecciones Asintomáticas, Klebsiella pneumoniae

## Abstract

El síndrome de la bolsa de orina púrpura (SBOP) es una condición infrecuente y generalmente asintomática, caracterizada por la coloración púrpura de la orina en pacientes portadores de sonda vesical, secundaria al metabolismo bacteriano del triptófano y la producción de pigmentos. Se describe el caso de un varón de 82 años, con antecedente de hiperplasia prostática benigna, hipertensión arterial y uso crónico de sonda Foley, quien acudió a consulta preoperatoria sin síntomas, presentando orina púrpura de 48 horas de evolución. El urocultivo evidenció crecimiento de *Klebsiella pneumoniae* productora de betalactamasa de espectro extendido. Dada la ausencia de síntomas urinarios o sistémicos, se optó por no administrar antibióticos y realizar únicamente el recambio del sistema de drenaje urinario. El paciente evolucionó favorablemente, con resolución del cambio de color. El SBOP, aunque llamativo, es un hallazgo benigno asociado a bacteriuria asintomática y su manejo debe evitar el uso innecesario de antibióticos.

## INTRODUCCIÓN

El síndrome de la bolsa de orina púrpura (SBOP) es una entidad clínica poco frecuente, caracterizada por una llamativa coloración púrpura del sistema de drenaje urinario, generalmente observada en pacientes con sondaje urinario crónico. Este fenómeno, aunque alarmante a la vista, suele ser benigno y asintomático en la mayoría de los casos, pero puede estar asociado con infecciones del tracto urinario (ITU) y condiciones debilitantes subyacentes que incrementan la morbilidad del paciente ^1^^-^^3^.

Fue descrito por primera vez en 1978, aunque se han sugerido observaciones clínicas similares desde el siglo XIX, como en el caso del rey Jorge III ^2^^,^^3^. La pigmentación característica se debe a la producción de compuestos derivados del metabolismo bacteriano del triptófano, lo que convierte al SBOP en una manifestación visible de un complejo proceso bioquímico subyacente ^1^^,^^3^^,^^4^.

Estudios recientes indican que el SBOP puede tener una prevalencia significativa en ciertos grupos de pacientes. Se ha reportado en hasta un 9,8% de los pacientes con cateterismo urinario prolongado ^2^^,^^3^. En hospitales geriátricos, hasta el 27% de los pacientes con demencia y portadores de sonda urinaria desarrollaron está patología ^1^. Los factores de riesgo frecuentemente asociados incluyen el sexo femenino, la edad avanzada, la demencia, el estreñimiento crónico, la deshidratación, la enfermedad renal crónica, la orina alcalina y una alta carga bacteriana en la orina ^1^^-^^3^. Esta condición predomina en mujeres y personas con estados de debilitamiento general, lo cual podría explicarse tanto por factores anatómicos como por la mayor frecuencia de cateterismo crónico en estas poblaciones ^2^^,^^3^^,^^5^.

Este caso resulta relevante por su presentación en un paciente varón asintomático portador de *Klebsiella pneumoniae* productora de betalactamasa de espectro extendido (BLEE), lo que resalta la naturaleza benigna del síndrome ^6^ y la importancia de un manejo conservador sin el uso de antibióticos.

## REPORTE DE CASO

Se presenta el caso de un paciente varón de 82 años, con antecedente de hiperplasia prostática benigna (HBP), hipertensión arterial controlada con losartán 50 mg/día y uso crónico de sonda vesical tipo Foley de silicona, calibre 18 Fr, durante aproximadamente tres años. No presentaba antecedentes de alergias medicamentosas ni consumo reciente de antibióticos. Refería el uso ocasional de laxantes osmóticos por estreñimiento funcional. El paciente mantenía ingesta hídrica aproximada de 1,5 L/día y residía en un entorno domiciliario, con cuidados familiares.

El motivo principal de consulta fue la observación de un cambio de coloración de la orina a tono púrpura en las últimas 48 horas. El paciente no refirió fiebre, disuria, dolor suprapúbico o lumbar. Al examen físico se encontraba afebril (36,7 °C), con presión arterial de 122/68 mmHg, frecuencia cardíaca de 76 lpm, frecuencia respiratoria de 18 rpm y saturación de oxígeno del 91% al aire ambiente, sin signos de irritación vesical ni compromiso sistémico. Se evidencio coloración púrpura tanto de la sonda vesical como de la bolsa colectora (figura 1), hallazgo compatible con SBOP.

**Figura 1:** Presencia de coloración púrpura en la orina contenida en la bolsa. (A) Bolsa recolectada el primer día de hospitalización; (B) bolsa recolectada el tercer día de hospitalización, previo al recambio del sistema de drenaje.

El uroanálisis mostró pH alcalino de 8,5, nitritos positivos, esterasa leucocitaria positiva, piuria leve y bacteriuria abundante. La función renal se encontró conservada (creatinina sérica de 1,0 mg/dL, depuración estimada > 60 mL/min). Se obtuvo una muestra de orina para análisis y cultivo. El urocultivo reveló crecimiento de *Klebsiella pneumoniae* productora de BLEE. A pesar de estos hallazgos microbiológicos, el paciente permaneció asintomático durante todo el seguimiento.

### Manejo

Ante la ausencia de síntomas urinarios o sistémicos, se decidió no administrar antibióticos, de acuerdo con las guías vigentes sobre el manejo de bacteriuria asintomática. Se realizó el recambio completo del sistema de drenaje urinario mediante técnica aséptica estándar incluyendo el reemplazo de la sonda Foley y la bolsa colectora. Asimismo, se brindó educación al paciente sobre medidas preventivas, tales como una adecuada higiene perineal, el manejo del estreñimiento, el mantenimiento de un adecuado flujo urinario y el recambio programado del sistema cada tres semanas.

### Evolución y seguimiento

En la figura 2 se presenta una línea de tiempo. Posteriormente, el paciente fue sometido al procedimiento quirúrgico programado, sin complicaciones. Durante el periodo posoperatorio y en controles subsiguientes, no se observó recurrencia de la coloración púrpura en la bolsa ni en la orina.

**Figura 2:** Flujograma de evolución y manejo del caso.

## DISCUSIÓN

La fisiopatología del SBOP (figura 3) se fundamenta en una compleja cascada metabólica del triptófano. Este aminoácido esencial es convertido en indol por la microbiota intestinal; posteriormente, el indol es absorbido a través de la circulación portal y metabolizado en el hígado a 3-hidroxiindol por el citocromo P450 2E1 (CYP2E1), el cual es luego sulfonado por la isoforma hepática SULT1A1 a indoxil sulfato ^7^^,^^8^. Este metabolito, excretado en la orina, puede ser descompuesto en el tracto urinario por fosfatasas y sulfatasas bacterianas de *Providencia stuartii*, *Proteus mirabilis*, *Escherichia coli*, *Pseudomonas aeruginosa*, *Klebsiella pneumoniae*, *Morganella* spp. y *Citrobacter* spp en un medio alcalino, originando índigo (azul) e indirrubina (rojo), y la mezcla de ambos pigmentos en las bolsas colectoras de PVC (cloruro de polivinilo) da como resultado la coloración púrpura característica ^7^^-^^9^. Cabe señalar que no todas las cepas bacterianas poseen igual actividad enzimática, por lo que la presencia de bacteriuria por sí sola no garantiza la aparición del síndrome ^8^^,^^9^. Adicionalmente, la elevada concentración de indoxil sulfato en pacientes con insuficiencia renal, debido a su escasa eliminación, potencia la disponibilidad del sustrato para la formación de pigmentos ^8^^,^^10^.

**Figura 3:** Fitopatología de la bolsa de orina púrpura. Se muestra cómo el triptófano de los alimentos es transformado por bacterias intestinales y luego por el hígado en un compuesto que se elimina por la orina. En presencia de ciertas bacterias y orina alcalina se forman pigmentos que reaccionan con el material de la bolsa colectora de orina produciendo el color púrpura característico.

Fuente: elaboración propia.

Entre los factores de riesgo más comúnmente asociados al SBOP se encuentran: sexo femenino, edad avanzada, estreñimiento crónico, demencia, postración, enfermedad renal crónica, dieta rica en triptófano, sonda vesical de permanencia prolongada y orina alcalina ^11^^-^^14^. La anatomía urinaria femenina, las alteraciones en el autocuidado favorecen la colonización bacteriana y la recurrencia de infecciones urinarias, especialmente en pacientes institucionalizados o postrados ^9^^-^^11^. La orina alcalina es determinante, aunque no indispensable, suele estar inducida por bacterias productoras de ureasa como *Proteus mirabilis, Providencia stuartii/rettgeri, Escherichia coli, Klebsiella pneumoniae y Pseudomonas aeruginosa*, todas ellas frecuentemente identificadas en cultivos urinarios de pacientes con SBOP ^7^^,^^8^. Asimismo, el estreñimiento retrasa el tránsito intestinal y prolonga la exposición del triptófano a la flora intestinal, facilitando su conversión a indol, por tanto, la producción de indoxil sulfato ^7^^,^^11^^,^^13^. De igual forma, la alta carga bacteriana urinaria se ha correlacionado con una mayor incidencia del síndrome ^8^^,^^9^^,^^12^.

El manejo del SBOP se basa en abordar los factores predisponentes y no en el tratamiento específico del cambio de coloración, dado que se trata de una entidad generalmente benigna; por ello, en la mayoría de los casos, la pigmentación desaparece tras resolver la causa subyacente ^7^^,^^8^^,^^10^. No obstante, debe realizarse un adecuado manejo de los dispositivos urinarios, incluyendo el recambio de la bolsa colectora, del catéter, así como la implementación de medidas de higiene y cuidados preventivos para evitar la colonización bacteriana ^7^^,^^9^^,^^12^. En pacientes con comorbilidades como enfermedad renal crónica, el monitoreo de los niveles de indoxil sulfato puede ser relevante dada su asociación con progresión de la nefropatía y eventos cardiovasculares ^8^^,^^12^. En casos severos con complicaciones infecciosas o signos de sepsis, se ha demostrado que la instauración temprana de terapia antibiótica apropiada en base a los factores de riesgo para resistencia bacteriana o según el resultado del antibiograma, permitirán reducir la morbilidad y mortalidad asociadas ^8^^,^^14^.

En línea con la guía de la Infectious Diseases Society of America (IDSA) ^15^ y el United States Preventive Services Task Force (USPSTF) ^16^, la bacteriuria en portadores de catéter vesical no debe tratarse con antibióticos en ausencia de síntomas, evitando resistencia antimicrobiana y efectos adversos, principios que son promovidos también por el Centers for Disease Control and Prevention (CDC) ^17^ y el National Institute for Health and Care Excellence (NICE) ^18^, que priorizan la reducción del uso innecesario de antimicrobianos y la prevención de resistencia.

En conclusión, el presente caso ilustra que el SBOP, pese a su apariencia alarmante, es una entidad benigna y generalmente autolimitada, asociada a bacteriuria asintomática en pacientes portadores de catéter vesical crónico. En este contexto, la identificación de *Klebsiella pneumoniae* productora de BLEE en ausencia de manifestaciones clínicas de infección urinaria refuerza la importancia de diferenciar la colonización bacteriana de una infección verdadera, evitando intervenciones terapéuticas innecesarias.

La resolución completa del cuadro tras el recambio del sistema de drenaje mediante técnica aséptica estándar, junto con la corrección de factores de riesgo, la implementación de medidas higiénicas y la educación al paciente, demuestra que un manejo conservador es suficiente cuando no existen síntomas urinarios o sistémicos.

Este enfoque es concordante con las recomendaciones de la IDSA, el USPSTF, el CDC y el NICE, que enfatizan la necesidad de restringir el uso de antibióticos a los casos sintomáticos, promoviendo el uso racional de antimicrobianos y la prevención de la resistencia bacteriana. En conjunto, este caso resalta la relevancia del juicio clínico, la vigilancia adecuada del paciente y las buenas prácticas en el manejo del cateterismo urinario como pilares para optimizar los resultados clínicos y prevenir complicaciones.
